# Towards New Uses of Botulinum Toxin as a Novel Therapeutic Tool 

**DOI:** 10.3390/toxins3010063

**Published:** 2011-01-12

**Authors:** Andy Pickett, Karen Perrow

**Affiliations:** Biologicals Science and Technology, Ipsen Biopharm Limited, Ash Road North, Wrexham, LL13 9UF, UK; Email: karen.perrow@ipsen.com

**Keywords:** clostridial neurotoxin, BoNT, engineering, targeting

## Abstract

The uses of botulinum toxin in the fields of neurology, ophthalmology, urology, rehabilitation medicine and aesthetic applications have been revolutionary for the treatment of patients. This non-invasive therapeutic has continually been developed since first discovered in the 1970s as a new approach to what were previously surgical treatments. As these applications develop, so also the molecules are developing into tools with new therapeutic properties in specific clinical areas. This review examines how the botulinum toxin molecule is being adapted to new therapeutic uses and also how new areas of use for the existing molecules are being identified. Prospects for future developments are also considered.

## 1. Introduction

Botulinum neurotoxins (BoNTs) have shown considerable clinical efficacy in treating a large range of disorders. Historically, the therapeutic utilization of BoNTs has progressed by administration for novel therapeutic interventions and future progress is likely to follow a similar path. Most recently, BoNT has been increasingly employed for example in the field of urology [1] and also BoNTs have been evaluated for the treatment of other new indications [2], for example painful keloid [3], diabetic neuropathic pain [4], refractory knee pain [5], trigeminal neuralgia trigger-zone application to control pain [6], scarring after cleft-lip surgery [7], cancer [8] and depression [9]. These all seek to use the toxin in the unmodified, *i.e.*, native form, to treat new indications. This review will, however concentrate on the adaptation of the BoNT molecule itself, engineered from the wild-type form to either alter the mode of action or to expand the range of therapeutic targets.

The clostridial neurotoxin family comprises seven BoNT serotypes (A-G), produced mainly by *Clostridium botulinum* and the tetanus neurotoxin (TeNT), produced by *Clostridium tetani* [10]. Although the BoNTs and TeNT function via a similar initial physiological mechanism of action, producing paralysis by inhibition of neurotransmission, they differ in their clinical response (flaccid paralysis for BoNT, rigid paralysis for TeNT), cellular targeting, substrate and duration of action. There are currently three major commercially available preparations of BoNT Type A (BoNT-A) toxins: Dysport^®^, Botox^®^ and Xeomin^®^, although several others are currently available in a few countries (e.g., Neuronox^®^, BTXA) and others are being developed (e.g., PurTox^®^), and one Type B toxin exists: Myobloc^®^[11]. In clinical treatment, BoNTs are traditionally administered into peripheral tissue, resulting in reversible blockade of the neuromuscular junction.

Here, we review the potential of modifying BoNTs to extend their range and number of therapeutic applications and to provide novel therapeutic tools for the future. We will summarize current knowledge of BoNT genetics and discuss the design of novel toxins for application in new therapeutic interventions. 

## 2. Genetic Organization of BoNT

BoNTs are synthesized as a single polypeptide chain comprising several domains with distinct functions that contribute to the mechanism of toxicity (discussed in further detail below). Other proteins produced from *Clostridium botulinum* form a complex with BoNT that may contribute to toxicity and the stability of the BoNT in the natural environment of food poisoning [12,13,14]. The potential of these accessory proteins to facilitate function within a therapeutic setting is not currently known.

**Figure 1:**
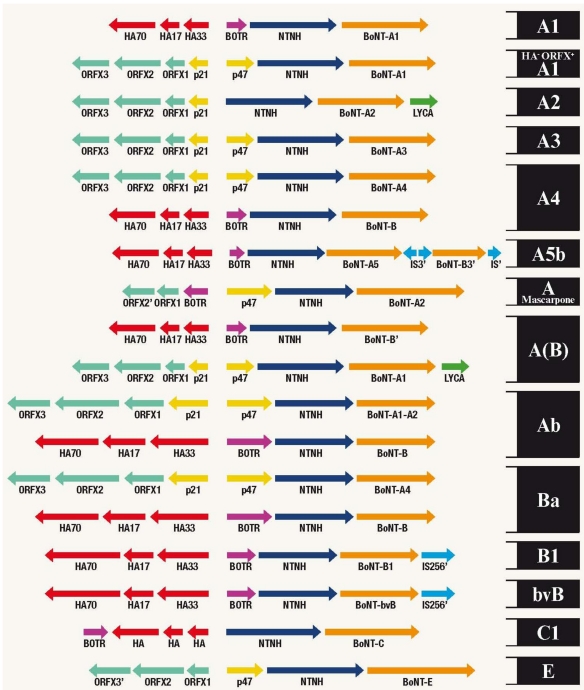
Arrangements of 18 BoNT gene clusters. Arrows indicate the respective positions and direction of genes identified in BoNT gene clusters. Gene nomenclature is provided beneath and strain identifications on the right. BoNT serotypes are indicated with a capital letter and subtypes by number. Silent genes are indicated in lower case. Partial genes are indicated with an apostrophe. Brackets indicate a second, partial sequence is expressed. HA, haemagglutinin; ORF, open reading frame; IS, insertion sequence; NTNH, non-toxic non-haemagglutinin. BOTR is a regulatory gene identified in the HA cluster [15,17,20,23,27,111,112].

Genes encoding the BoNTs, other members of the protein complex and genes that regulate expression of the toxin, are grouped in clusters. Great variation exists between BoNT gene clusters (Figure 1). Unique clusters containing the BoNT-A gene are designated as subtypes A1-A5 [15], with new subtypes of the BoNTs regularly being identified [16,17,18]. 

**Figure 2:**
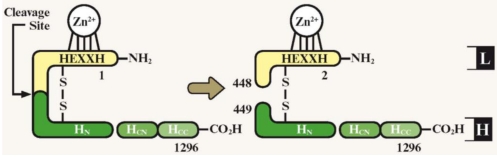
Schematic representation of BoNT-A domain structure. Proteolytic cleavage activates BoNT, yielding a di-chain protein joined by a disulfide bond. The heavy chain (green) is composed of domains: H_N_ and the H_C_, which is involved in translocation of the light chain (L, yellow). The H_C_ is further divided into two subdomains: H_CN_ and the H_CC_, which is involved in neurospecific binding. The light chain possesses endopeptidase activity, with a zinc-binding motif (HEXXH). Numbers indicate amino acid residues within the complete neurotoxin gene [8]. H: Heavy chain; H_N_: Heavy chain N-terminal fragment; H_CN_: Heavy chain C-terminal fragment, N-terminal subdomain; H_CC_: Heavy chain C-terminal fragment, C-terminal subdomain; L: Light chain. Figure adapted from [8], permission obtained.

Both the BoNT complex and the functional domains of the toxic BoNT peptide are modular in nature (Figure 2). This is a reflection of the arrangement of the genes in the cluster, making the toxin amenable to genetic engineering. The prospect of genetic engineering is further enriched by the functional domains of the BoNTs themselves being arranged in a linear fashion, so that domains at either end of the molecule can be manipulated with minimal impact on the central domain [19].

Genomes from several *C. botulinum* strains have now been sequenced [20], revealing the genetic diversity of BoNT [21]. Comparison of BoNT nucleotide and amino acid sequences, which differ by up to 8% and 16%, respectively [21], has been used to predict differences in substrate binding and catalysis [22]. In contrast, the number and composition of reading frames within gene clusters (Figure 1) [15,16,23], and their location, on chromosomes, plasmids or bacteriophages, all vary in a serotype-specific manner [23,24,25,26,27]. 

## 3. Membrane Binding Initiates BoNT Mechanism of Action

All BoNT neurotoxins are synthesized initially as a ~150 kDa single chain, which is cleaved by an (unidentified) clostridial enzyme to form the active BoNT complex. This comprises a ~50 kDa light chain, which is a zinc-dependent endopeptidase, and a ~100 kDa heavy chain; the two are linked by a single disulfide bond [10]. The heavy chain, particularly the C-terminal domain (H_C_), mediates uptake of the toxin into the neuron, by binding to recycling synaptic vesicles in a stimulation-dependent manner [28,29]. Uptake is selectively directed to neuronal targets by specific high-affinity binding domains on the heavy chain, which interact with protein and ganglioside components of the cell membrane in a serotype-dependent manner (Table 1) [30,31].

The highly-expressed gangliosides [32] represent the lower-affinity receptors [33] responsible for accumulating BoNT on the neuronal membrane. Upon stimulation of the neuromuscular junction and subsequent recycling of synaptic vesicles, the amino-terminal intra-vesicular domain of the protein receptor is exposed, allowing toxin binding and endocytosis [34,35]. 

Comparison of the crystal structures of BoNT-A and BoNT-F receptor-binding domains reveals the heavy chain folds of BoNT-A and BoNT-F are similar, except for the region implicated in neuron binding [36]. A similar trend has also been reported for BoNT-B and BoNT-G, with differences in crystal structure explaining both ganglioside- and protein-receptor specificities [37]. Understanding such interactions in the future may allow BoNT engineering to bind non-neuronal cells.

Despite the variation exhibited by this region, a single, highly conserved ganglioside-binding motif E(D)…H…S(G)XWY…G(S) has been identified in the H_C_ of BoNT-A, BoNT-B, BoNT-E, BoNT-F and BoNT-G [38,39,40]. An additional carbohydrate-binding domain has also been identified in the H_C_ of BoNT-D [41]. These motifs determine the serotype specificity of the BoNT/carbohydrate interaction (Table 1) [37,39,42], as modification of residues within these sites is reported to alter the binding, uptake and toxicity of BoNT [38,39,40,41,42,43,44,45,46,47,48,49]. In particular, substitutions have been identified that enhance BoNT binding and toxicity [43], an effect that has been proposed to increase potency for use in the clinic [44]. 

**Table 1:** Binding and catalytic targets of BoNT serotypes serotypes (A-G). TeNT, tetanus toxin; SV2A, B and C, synaptic vesicle glycoprotein 2A/B/C, Syn I and II, synaptotagmins I/II, SNAP-25, synaptosomal-associated protein 25; VAMP, vesicle-associated membrane protein; ThyI, Thy-1 cell surface antigen [33,37,41,42,44].

Serotype	Cellular binding receptors		Catalytic target
	Carbohydrate	Protein	
A	GD1a, GD1b, GT1b, GQ1b	SV2A, B and C	SNAP-25
B	GD1a, GD1b, GT1b	Syn I and II	VAMP
C1	GD1a, GD1b ,GT1b		SNAP-25 and syntaxin
D	GT1b, GD2		VAMP
E	GD1a, GT1b, GQ1b	Glycosylated SV2A and B	SNAP-25
F	GD1a, GD1b, GT1b	SV2	VAMP
G	GT1b	Syn I and II	VAMP
TeNT	GT1b, GD1b GM1a GD3	ThyI	VAMP

## 4. Modifying the Binding Domain of BoNT to Retarget the Native Catalytic Domain

The light chain is catalytically active if introduced to non-neuronal cells via permeabilization [45,46], microinjection [47], transfection with the gene encoding the light chain [48] and modification of the BoNT-binding domain [49,50,51]. Retargeting the neuronal specificity of binding is a key aim of BoNT engineering, as delivery of the light chain, or non-native proteins, to the cytosol of non-neuronal cells may allow, for example, treatment of non-neuronal secretory diseases.

Several approaches have been used to target non-neuronal cells with BoNT. Co-application with lipid-based DNA transfection reagents resulted in BoNT-A activity in non-neuronal cell lines that are resistant to the toxin when applied alone [52]. Other approaches have exploited elements of the actual BoNT, substituting single amino acids [53] and whole domains of the toxin, summarized in Table 2 and discussed in detail here.

Generating chimeric proteins from BoNT has combined desired BoNT characteristics within one toxin. A chimera composed of the light chain and N-terminal heavy chain (H_N_) of BoNT-E with the heavy chain of BoNT-A (chimera E/A) displayed the rapid uptake and block of neuromuscular transmission exhibited by BoNT-E [54]. This chimera has been used to target a sensory relay centre implicated in pain mediation that is resistant to both parent toxins [55]. 

Conjugating light chain catalytic domains of BoNT to cell-binding domains of non-toxic proteins has rendered refractory cells sensitive to the toxin. A heterodimer (termed LH_N_) consisting of the light chain and heavy chain N-terminus (H_N_) is of particular interest for the design of novel therapeutic conjugates as this still contains both cell-membrane transportation and catalytic actions of the original BoNT molecule [19,51,56]. Recombinant studies have produced conjugates of the LH_N_ heterodimer containing fragments of BoNT-A, BoNT-B and BoNT-C [57]. The LH_N_ of BoNT-A has also been conjugated to lectin from *Erythrina cristagalli*, which binds to galactose-containing carbohydrates found on the surface of nociceptive afferents, to target pain signalling *in vivo* [49]. Chaddock *et al.* showed that the same BoNT-A LH_N_ conjugated to wheat germ agglutinin inhibited neurotransmitter release from neuronal cell lines normally resistant to the neurotoxin [50]. Using a similar approach, this BoNT-A LH_N_ was targeted to neuroblastoma cells that are normally refractory to the fragment by conjugation to nerve growth factor, inhibiting neurotransmitter release [51].

**Table 2:** Summary of modifications to the BoNT-binding domain to generate molecules with new therapeutic potentials as indicated. SNAP-25, synaptosomal-associated protein 25; C2IN, enzymatically-inactive binding domain C2IN of BoNT-C2; GFP, green fluorescent protein; LH_N_, heterodimer consisting of the light chain and amino-terminal domain of the heavy chain; PEP-1, carrier protein.

Modification to Binding Domain	Effect	Therapeutic Potential	Reference
BoNT-A/E chimera	SNAP-25 cleavage similar to BoNT-A	Similar to that of BoNT-A	[54,55]
BoNT-E/A chimera	Rapid uptake similar to BoNT-E	More persistent muscle weakening, targeted pain mediation	[54,55]
C2IN-streptavidin	Delivery of biotinylated molecules	Drug delivery	[61]
S6 peptide	Delivery of small molecules	Drug delivery	[60]
Fluorescent proteins e.g., GFP	Tracer molecules	Analysing neuronal circuit plasticity	[64,65]
Drug activating enzyme	Drug activation	Chemotherapy	[67]
Poly-lysine	DNA delivery	Gene therapy	[68,70]
Lectin	Binds nociceptive afferents	Targeted pain medication	[49]
Wheatgerm agglutinin	Targeted light chain to neuronal cells	Inhibited refractory neurotransmitter release	[50]
Nerve growth factor	Targeted LH_N_ neuronal cells	Inhibited refractory neurotransmitter release	[51]
Epidermal growth factor	Targeted epithelial cells	Inhibited mucus secretion	[58]
Addition of PEP-1 peptide	Penetrated skin	Novel administration technique	[88]

Providing proof-of-principle that a retargeted BoNT derivative can prevent secretion in non-neuronal cells, Foster *et al.* conjugated BoNT-C LH_N_ and epidermal growth factor (EGF) to inhibit the secretion of mucus from epithelial cells that are refractory to the LH_N _alone [58]. This BoNT-EGF derivative has the potential to treat the hyperactive mucus secretion associated with asthma and chronic obstructive pulmonary disease and the approach could be used, in concert with a BoNT, to relieve overactive smooth muscle contraction that contributes to such respiratory diseases [59].

## 5. Employing the BoNT-Binding Domain to Deliver Non-Native Proteins

Chimeric BoNTs have also been developed that employ the cellular binding activity of BoNT as a targeting moiety to deliver the activity of a conjugated, non-native protein. Removing or inactivating the catalytic light chain domain of BoNT is required in order to prevent the blockade of neurotransmission. Catalytically-inactive BoNTs are therefore proposed to have prolonged cellular uptake, in contrast to active BoNTs that disable their own uptake [60]. 

Genetic fusion of the enzymatically-inactive binding domain C2IN of BoNT-C2 to streptavidin allowed delivery of biotinylated molecules into the cytosol of mammalian cells [61]. Full-length BoNTs containing activating mutations have been fused to an S6 peptide sequence, allowing the attachment and delivery of a fluorescent small-molecule to the target cell cytoplasm [60]. Conjugating BoNT to such carrier proteins exponentially increases the number of potential targeted cargos, providing vehicles for future targeting of small molecule drugs.

Various tracer proteins have also been conjugated to BoNT peptides, including those containing inactivating mutations [62]. Conjugates with β-galactosidase [63] and fluorescent proteins [62,64,65] have revealed BoNT and TeNT distribution profiles, allowing mapping of neuronal circuits [65] and analysis of neuronal circuit plasticity after traumatic injury or neurodegenerative diseases [66]. 

The BoNT cellular-binding activity has also been proposed to deliver an enzyme to cancer cells that activates a prodrug, administered systemically [67]. However, catalytically active BoNTs, which are proposed to enhance tumor perfusion and subsequent access of cytotoxic agents and oxygen in order to potentiate radiotherapy, may be more appropriate for cancer therapy [8]. 

As TeNT has a similar modular arrangement of functional domains as BoNT, advances in TeNT engineering may also be applied to BoNT. For example, the binding domains of both BoNT and TeNT have been utilized to deliver DNA to target cells and enhance targeting of other transfection methods. The TeNT heavy chain fragment has been conjugated to poly-lysine, which has a high capacity to bind DNA, allowing transfection of a range of neuronal cell lines [68,69]. This TeNT-poly-lysine derivative has also been shown to enhance adenoviral infection of primary neuronal cultures while increasing neuronal specificity of transfection [70]. TeNT and BoNT heavy chain have also been added to liposomes, targeting gene delivery *in vitro* [71].

Recent studies with recombinantly produced BoNT domains show that proteins can be assembled by non-chemical linking, using tagging with helical motifs from the family of soluble N-ethylamide-sensitive factor attachment protein receptor (SNARE) proteins. This may potentially be exploited to use the BoNT-binding domain to deliver future therapeutics or other cargo into neurons, or to facilitate re-targeting of the light chain [72].

## 6. Modifying the BoNT Active Site to Target Non-Native Substrates

BoNT-A has the potential to inhibit the release of multiple, but not all, neurotransmitters [73,74]. Thus, in addition to neuronal specificity being conferred by the binding domain, a degree of specificity is also bestowed by the catalytic domain. Manipulation of the BoNT light chain catalytic domain may therefore also be important when designing BoNT to target non-neuronal cells or in modifying the targets within neuronal systems. 

Once taken up into the neuron, BoNT light chain cleaves members of the SNARE family of proteins which mediate docking of synaptic vesicles with the neural cell membrane [75]. The SNARE proteins are a large family: 36 members have so far been identified in mammalian cells [76], including a 25 kDa synaptosomal-associated protein (SNAP-25), synaptobrevin (also called vesicle-associated membrane protein (VAMP) and syntaxin. Cleavage and inactivation of SNAREs by the BoNTs results in inhibition of neurotransmission and concomitant prevention of further toxin uptake. BoNT-A, BoNT-C and BoNT-E cleave SNAP-25 while BoNT-B, BoNT-D, BoNT-F and BoNT-G cleave proteins of the VAMP family (Table 1); [44]. BoNT-C also cleaves syntaxin [44]. 

In addition to neurotransmission, SNAREs have been implicated in non-neuronal secretory processes. The effectiveness of BoNT on these processes is determined by both the SNARE member mediating secretion and the BoNT serotype. For example, BoNT-A is reported to inhibit insulin secretion from permeabilized β-cell lines, as this process is mediated by SNAP-25 [45]. In contrast, insulin-stimulated uptake of glucose to permeabilized adipocytes, which is mediated by SNAP-23, is resistant to BoNT-A [77]. Engineering BoNT to modulate insulin signalling may allow application of BoNT in diabetes therapy. Moreover, this approach may target non-neuronal SNAREs in a variety of non-neuronal secretory disorders.

Regions of BoNT involved in SNARE recognition and cleavage have been identified [78,79], including the residues directly involved in catalysis [80], although residues both near and distal to the active site are reported to be important in their proteolytic action [81,82]. Primary sequences are also now available for 36 human SNAREs [76] and mapping the sites cleaved by members of the clostridial neurotoxins family has been completed (Table 3) [83,84]. This indicates that specific residues on both the BoNT and the substrate are involved in catalysis.

The BoNT regions implicated in catalysis are supported by crystal structures of inactive BoNT-A light chain bound to SNAP-25, confirming exosite and active site interactions [85]. Correlating structural data with sequence identity has explained serotype-specificity of substrate binding and catalysis, allowing substrate prediction in newly discovered BoNT species [22]. Structure-function relationships may allow directed engineering of BoNT to provide alternatively targeted molecules.

Although the minimal substrate size of BoNT-A required for catalysis is relatively large, only a few non-conserved residues proximal to the active site have been shown to influence catalysis of the substrate [82]. Such residues are sensitive to subtle changes in amino acid composition; substituting one positively-charged residue (arginine) for another (lysine) in BoNT-A*(R230K)*, for example, abolishes activity [82]. Taken together, these data have allowed rational, directed approaches to retarget the catalytic activity of BoNT and cleave non-neuronal SNARE proteins. 

In order to target non-neuronal SNARE proteins, Chen *et al.* [53] targeted position 224 within the catalytic site of BoNT-E, which specifically cleaves the neuronal SNARE, SNAP-25. In addition to cleaving SNAP-25, the engineered BoNT-E *(K224D)* cleaved non-neuronal SNAP-23, at a similar rate to that at which the wild-type toxin cleaves its native target [53].

**Table 3:** SNARE isoform sequences indicating BoNT cleavage sites. Amino acid residues are indicated in lower case. Cleavage sites are indicated by a dashed line between specific amino acids of the toxin sequence. Highlighted residues indicate non-conserved mutations at or around cleavage sites. SNARE, soluble N-ethylamide-sensitive factor attachment protein receptor; VAMP, vesicle-associated membrane protein; SNAP, synaptosomal-associated protein. Table adapted from [94] copyright retained by Inderscience.

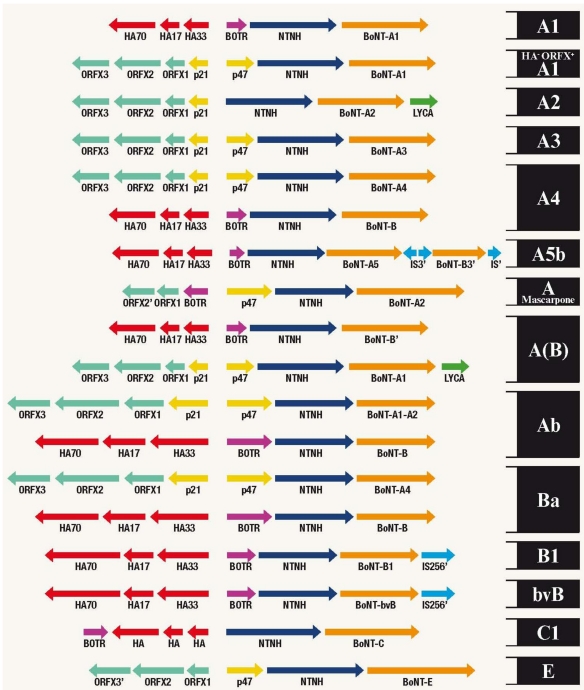

Catalytically-retargeted BoNT may be applied clinically in the future to target non-neuronal cells in concert with novel administration technologies, such as iontophoresis [86], nasal inhalation [87], and fusion proteins capable of penetrating the skin [88]. A recent report also demonstrated intranasal administration of BoNT-A H_C_ in a ‘nanometer-sized hydrogel’ (nanogel) [89], allowing binding and penetration of nasal epithelial cells. Once the H_C_ was released from the nanogel, the peptide was taken up by nasal dendritic cells, without accumulation in the brain [89]. Several patents for novel BoNT administration have also been filed (Table 4), including skin disruption with transdermal patches [90], release of polymeric microspheres from implants [91] and toxin application in phospholipid micelles [92] and solvents [93].

**Table 4:** Recent patents involving modifications to botulinum toxin molecules intended to generate new molecules with additional therapeutic potentials and targets.

Invention	Author(s)	Patent No.	Reference
Application via transdermal patches	Donovan	US20017758871	[90]
Application via skin disruption	Donovan	US20017758871	[90]
Application in polymeric microsphere-containing implant	Donovan	US20080028216	[91]
Application in phospholipid micelles	Modi	US20080220021	[92]
Application in non-polar solvent	Petrou and Vedra	US20090304747	[93]
PEGylated mutated BoNT	Frevert and Specht	EP1834962	[107]
Formulations for oral administration	Donovan	US20040086532	[108]
Biodegradable neurotoxin implants	Hughes and Orest	US20050232966	[109]
Leucine-based motif and Clostridia neurotoxins	Steward *et al.*	US20080177041	[110]

## 7. Modifications to BoNT Duration of Action

In most clinical applications, the actions of BoNT are temporary, lasting from several days (BoNT-E) to months (BoNT-A) [19]. The duration of action is also highly species-dependant: BoNTs display the shortest action at the mouse neuromuscular junction, followed by rat, then human [94]. Adults are also reported to display greater sensitivity than juveniles [95], which may arise from differences in the abundance of motor endplates. Although the reversibility of BoNT activity can be considered desirable, for altering muscle involvement or fine-tuning aesthetic effects, a long duration of action is also advantageous, to reduce administration frequency. 

Differences in duration of the neuroparalytic effects between BoNT-A and BoNT-E may be due to the nature of the cleavage of the target protein [96]. Both cleave the SNARE complex component SNAP-25; BoNT-A truncates this protein by removal of 9 amino acids from the C-terminal end and BoNT-E cleaves 26 residues from the C-terminal end. Potentially the BoNT-A truncated SNAP-25 can still interact with the other SNARE proteins to form non-functioning SNARE complexes and so prevent exocytosis. Newly synthesised SNAP-25 cannot then interact with these blocked complexes, so causing paralysis to persist. Conversely, BoNT-E cleaves the SNAP-25 in the cell, causing paralysis, but the cleavage product does not associate with the other SNARE proteins in the same way, and newly synthesised intact SNAP-25 can then interact as normal to form the SNARE complex and the neuromuscular function is therefore resumed earlier than when BoNT-A is present.

The duration of action is also proposed to be determined by the intracellular persistence of the light chain [97], which in turn results from the cellular distribution of the peptide. Fernández-Salas *et al.* [98,99] compared the distribution of green fluorescent protein (GFP) and GFP conjugated to BoNT-A light chain when expressed in neuroblastoma cell lines. Cells expressing unconjugated GFP exhibited diffuse fluorescence throughout the cell. In contrast, cells expressing the GFP- light chain conjugate displayed a discrete pattern of fluorescence distributed in a manner resembling a cell outline, which co-distributed with SNAP-25 [98], indicating BoNT-A light chain directed distribution away from the cytosol. They showed that the duration of action of light chain from BoNT-A, BoNT-B and BoNT-E corresponded with cellular distribution. In particular, a cytosolic distribution was shown to correlate with a short duration of action [99]. Building on these data, Fernández-Salas *et al.* reported the catalytic light chain domain of BoNT-A, which is not involved in the neuron-specific endocytotic cellular binding, contains signals in the N- and C-termini that are required for plasma membrane binding [98]. Consistent with this, a chimeric BoNT composed of the light chain N-terminal (L_N_) of BoNT-A and H_C_ of BoNT-E (chimera AE) possessed similar persistence of SNAP-25 cleavage *in vitro* and neuromuscular block *in vivo* to BoNT-A, suggesting regions involved in duration of action are located in the L_N_. Such regions may be amenable to direct engineering in the future to extend the duration of toxin action in the clinic. In the past, many genetic approaches have been hindered by the number of clostridial species that are amenable to genetic manipulation, although novel techniques have recently been developed.

Studying and potentially altering potentially altering the duration of action of the BoNTs is particularly relevant, as there may be a limited amount of toxin that can enter the cell. The toxins mode of entry into the cell is by exploiting the synaptic vesicle pathway that couples exocytosis with endocytosis of synaptic vesicles. Binz and Rummel [28] claim that, as toxins interfere with this machinery and disrupt the cycle by cleavage of the proteins involved in this process, they will prevent the cycle from functioning and therefore will prevent their own further uptake into the cell. On this premise, there is a limited amount of toxin that can get into the cell and its longevity inside the cell therefore governs the length of the effect of the toxin. However, recent studies have indicated that the endocytosis of synaptic vesicles is still occurring in toxin poisoned cells [100,101]. These papers describe the potential for a modified BoNT (encompassing a cargo of 10 kDa amino dextran molecule coupled to a non-toxic recombinant heavy chain) to be transported into the cell as a drug delivery vehicle for rescue of botulinum toxin poisoned synapses as a countermeasure for botulism victims. Overall therefore, the exact mechanism of persistence of BoNT activity remains to be elucidated.

## 8. Alternative Methods of Modifying BoNT

Exploitation of *C. botulinum* sequence data has been hindered by the number of mutations generated for functional genomic studies, owing to a lack of basic molecular biology tools required for directed mutation. Targeted inactivation of clostridial genes has been almost exclusively limited to single crossover knockouts via integration through homologous recombination of a replication-defective plasmid [102]. However, capitalizing on these mutants has been restricted by the unstable integration of a plasmid with the chromosome. To address these issues, recombination-independent strategies have been devised that utilize a retargeted group II intron. One of these, “ClosTron”, allows systematic inactivation of genes to evaluate their function [103,104]. 

ClosTron provides the facility for positive selection of desired mutants, which are highly stable and reproducible, expanding the current options for functional genomic studies in clostridia. Although use in BoNT engineering is still in its infancy, ClosTron has huge potential. To date, ClosTron has been utilized to generate a non-toxigenic mutant strain of *C. botulinum* [105] and inactivate restriction endonuclease activity to allow transformation with unmethylated DNA as efficiently as with methylated DNA in *C. acetobutylicum* [106].

## 9. Summary and Conclusion

BoNTs have unique and well-characterized structural properties that make them particularly well suited to engineering for therapeutic use. The potential therapeutic capacity of BoNT is being realized through characterization and genetic engineering to alter the binding, catalysis and duration of the toxin, allowing specific targeting and therapeutic tailoring. Structure-function relationships allow rational design of catalytic specificity, allowing application of BoNT to numerous SNARE-mediated secretory processes, including those involved in diabetes [45,77], respiratory disorders [53] and processes mediating immune and inflammatory disorders [58]. Genetic engineering and functional studies are revealing the true potential of BoNT and have already expanded and diversified the potential therapeutic applications of BoNT, with each development yielding more ways to exploit and capitalize on the actions of this remarkable toxin.
